# Active YAP promotes pancreatic cancer cell motility, invasion and tumorigenesis in a mitotic phosphorylation-dependent manner through LPAR3

**DOI:** 10.18632/oncotarget.5935

**Published:** 2015-09-30

**Authors:** Shuping Yang, Lin Zhang, Vinee Purohit, Surendra K. Shukla, Xingcheng Chen, Fang Yu, Kai Fu, Yuanhong Chen, Joyce Solheim, Pankaj K. Singh, Wei Song, Jixin Dong

**Affiliations:** ^1^ Department of Oncology, Shandong Provincial Hospital affiliated with Shandong University, Jinan, Shandong, P. R. China; ^2^ Department of Radiation Oncology, Qilu Hospital of Shandong University, Jinan, Shandong, P. R. China; ^3^ Eppley Institute for Research in Cancer, Fred and Pamela Buffett Cancer Center, Omaha, NE, USA; ^4^ Department of Biostatistics, School of Public Health, Omaha, NE, USA; ^5^ Department of Pathology and Microbiology, University of Nebraska Medical Center, Omaha, NE, USA

**Keywords:** Hippo-YAP pathway, PDAC, cell motility, LPAR3, mitotic phosphorylation

## Abstract

The transcriptional co-activator Yes-associated protein, YAP, is a main effector in the Hippo tumor suppressor pathway. We recently defined a mechanism for positive regulation of YAP through CDK1-mediated mitotic phosphorylation. Here, we show that active YAP promotes pancreatic cancer cell migration, invasion and anchorage-independent growth in a mitotic phosphorylation-dependent manner. Mitotic phosphorylation is essential for YAP-driven tumorigenesis in animals. YAP reduction significantly impairs cell migration and invasion. Immunohistochemistry shows significant upregulation and nuclear localization of YAP in metastases when compared with primary tumors and normal tissue in human. Mitotic phosphorylation of YAP controls a unique transcriptional program in pancreatic cells. Expression profiles reveal LPAR3 (lysophosphatidic acid receptor 3) as a mediator for mitotic phosphorylation-driven pancreatic cell motility and invasion. Together, this work identifies YAP as a novel regulator of pancreatic cancer cell motility, invasion and metastasis, and as a potential therapeutic target for invasive pancreatic cancer.

## INTRODUCTION

Pancreatic cancer (mainly pancreatic ductal adenocarcinoma-PDAC) is one of the deadliest human cancers, with a five-year survival rate about 4%. This is largely due to the metastasis/advanced state of the disease at the time of diagnosis in most patients [1]. There is no widely used method for the early detection or effective treatment for pancreatic cancer. Surgery, radiation therapy, and chemotherapy only mildly extend survival and/or relieve symptoms, but seldom cure [1, 2]. Therefore, identification of novel targets and developing new therapeutic approaches has great significance for patients with invasive pancreatic cancer.

Pioneer genetic screens in the fruit fly *Drosophila* searching for growth regulators have discovered the Hippo signaling pathway. The Hippo pathway is highly conserved and its dysregulation contributes to cancer development [3-7]. In mammals, the Hippo core comprises the tumor suppressors Mst1/2 (mammalian sterile-20 like protein 1/2), WW45 (WW domain containing protein), Lats1/2 (large tumor suppressor 1/2) and Mob1 (Mps one binder protein 1). Protein kinases Mst1/2 associate with WW45, which phosphorylates and activates Lats1/2 and the adaptor protein Mob1. Activated Lats1/2 phosphorylates and inactivates the downstream effectors YAP/TAZ (Transcriptional coactivator with PDZ binding domain) by sequestering them in the cytoplasm and degrading them. Without inhibition through Hippo signaling, YAP/TAZ translocate into the nucleus, where they bind to transcription factors TEAD1-4 (TEA-domain containing) and induce expression of genes that promote cell proliferation and inhibit apoptosis.

Recent genetic mouse models and studies with cancer patients have firmly demonstrated the critical roles of Hippo-YAP signaling in tumorigenesis [6-8]. Although many cues or regulators activate or inhibit the Hippo pathway, YAP remains the most critical downstream effector of the Hippo pathway in tumorigenesis [6, 8, 9]. YAP is located in the 11q22 amplicon, which is involved in a variety of human cancers [10]. The oncogenic role of YAP has been extensively confirmed in many types of human malignancies [8]. We and others have shown that YAP is hyperactivated or overexpressed in pancreatic cancer patient tumor samples [11, 12], and YAP is required for Kras-driven pancreatic cancer development [12]. Furthermore, YAP activation is an important mechanism to drive pancreatic tumor growth in Kras-independent PDAC recurrence [13]. Over 90% of cancer-related deaths are due to metastasis, however, the functional role of YAP in pancreatic cancer cell motility and the metastasis of this deadly malignancy is still unclear. Here, we explored the biological significance of YAP in pancreatic cancer cell motility and invasion (critical processes for metastasis) and determined the clinical relevance of YAP in PDAC metastasis. Our data identify YAP as a novel regulator in the metastasis, as well as the tumorigenesis, of pancreatic cancer.

## RESULTS

### YAP promotes pancreatic cancer cell migration and invasion in a mitotic phosphorylation-dependent manner

**Figure 1:**
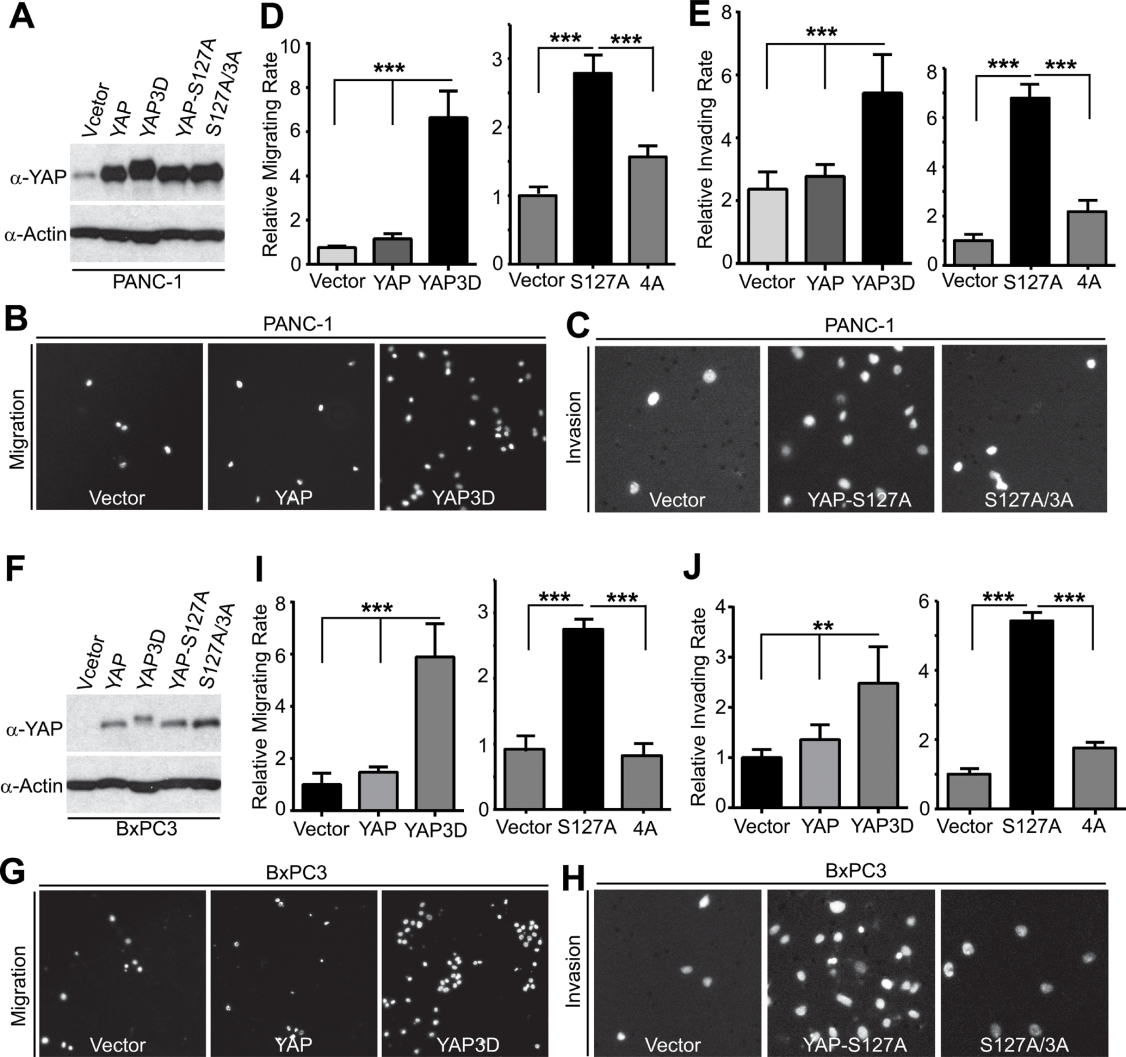
YAP promotes migration and invasion in a mitotic phosphorylation-dependent manner in pancreatic cancer cells **A.**, Establishment of PANC-1 cell lines stably expressing vector, YAP, YAP3D, YAP-S127A, and YAP4A (S127A/3A). 4A: S127A/T119A/S289A/S367A; 3D:T119D/S289D/S367D. **B.**-**E.**, Cell migration (B and D) and invasion (C and E) assays with PANC-1 cells expressing various YAP constructs. **F.**, Establishment of BxPC3 cell lines stably expressing vector, YAP, YAP3D, YAP-S127A, and YAP4A (S127A/3A). 4A: S127A/T119A/S289A/S367A; 3D:T119D/S289D/S367D. **G.**-**J.**, Cell migration (G and I) and invasion (H and J) assays with BxPC3 cells expressing various YAP constructs. Cell migration assays with Transwell and invasion assays with Matrigel were performed as we have previously described [14]. Migrated and invaded cells were stained with DAPI, and representative fields are shown. Data are expressed as the mean ± s.e.m. of three experiments. ***: *p* < 0.001, **: *p* < 0.01 (*t*-test).

**Figure 2:**
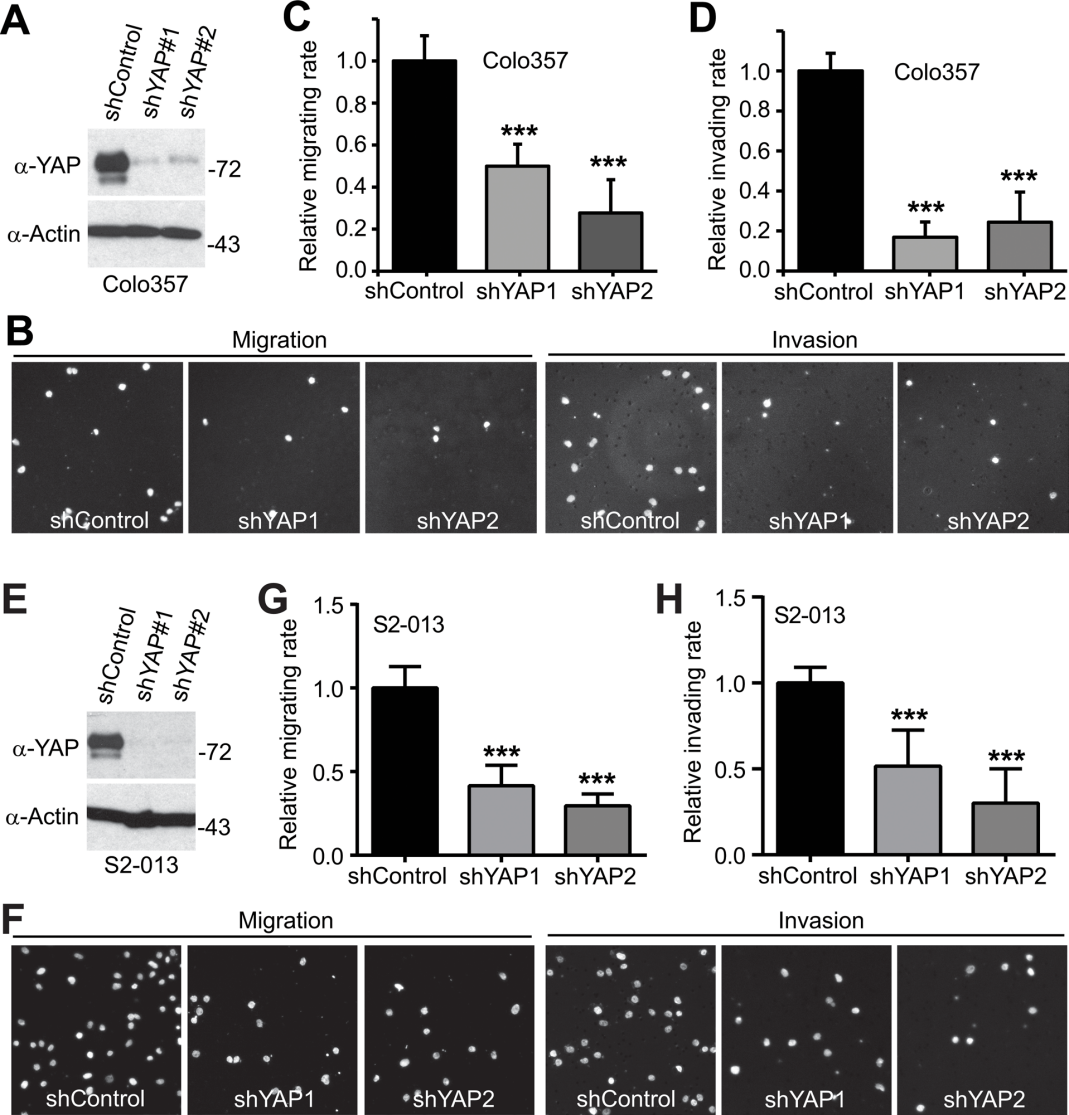
Knockdown of YAP impairs migration and invasion in pancreatic cancer cells **A.**, Establishment of cell lines stably expressing shRNA vector, and shRNAs against YAP (shYAP1 and shYAP2) in Colo357 cells. **B.**-**D.**, Cell migration and invasion assays with Colo357 cells established in A. **E.**, Establishment of cell lines stably expressing shRNA vector, and shRNAs against YAP (shYAP1 and shYAP2) in S2-013 cells. **F.**-**H.**, Cell migration and invasion assays with S2-013 cells established in E. Migrated and invaded cells were stained with DAPI, and representative fields are shown. Data are expressed as the mean ± s.e.m. of three experiments. ***: *p* < 0.001 (*t*-test).

YAP has been shown to stimulate pancreatic cancer cell proliferation [12, 13]; however, it is not known whether YAP promoted cell migration, invasion, and metastasis of pancreatic cancer. Considering clinical features (early stage invasion and metastasis) of PDAC, we explored the role of YAP and its mitotic phosphorylation [14] in pancreatic cancer cell motility. We have established cell lines stably expressing vector, YAP, YAP3D (a mitotic phosphorylation mimetic mutant, T119D/S289D/S367D), YAP-S127A (a Hippo-phosphorylation-deficient hyperactive mutant) and YAP-S127A/3A (4A, a mitotic phosphorylation-deficient mutant) in PANC-1 cells, which express relatively low levels of YAP (Figure 1A). Expression of wild type YAP was not sufficient to induce migration and invasion in these cells; however, both YAP-S127A and YAP3D robustly promoted migration and invasion (Figure 1B-1E). Interestingly, mutating mitotic phosphorylation sites to alanines (YAP4A) largely suppressed YAP-S127A-driven migration and invasion in PANC-1 cells (Figure 1C-1E). Similar results were obtained in BxPC3 cells (Figure 1F-1J). We also examined the result of loss of function of YAP in YAP-high pancreatic cancer cells (Colo357 and S2-013) (Figure 2A, 2E). No significant cell cycle alterations and signs of apoptosis were detected in YAP knockdown cells and these cells proliferated normally when compared to their corresponding control cells (see below and data not shown). However, shRNA-mediated YAP knockdown significantly impaired migration and invasion in both Colo357 and S2-013 cells (Figure 2). Thus, these data reveal a novel mechanism whereby hyperactive YAP-S127A promotes cell motility and invasion in a mitotic phosphorylation-dependent manner in pancreatic cancer cells.

### Mitotic phosphorylation of YAP is required for anchorage-independent growth

**Figure 3:**
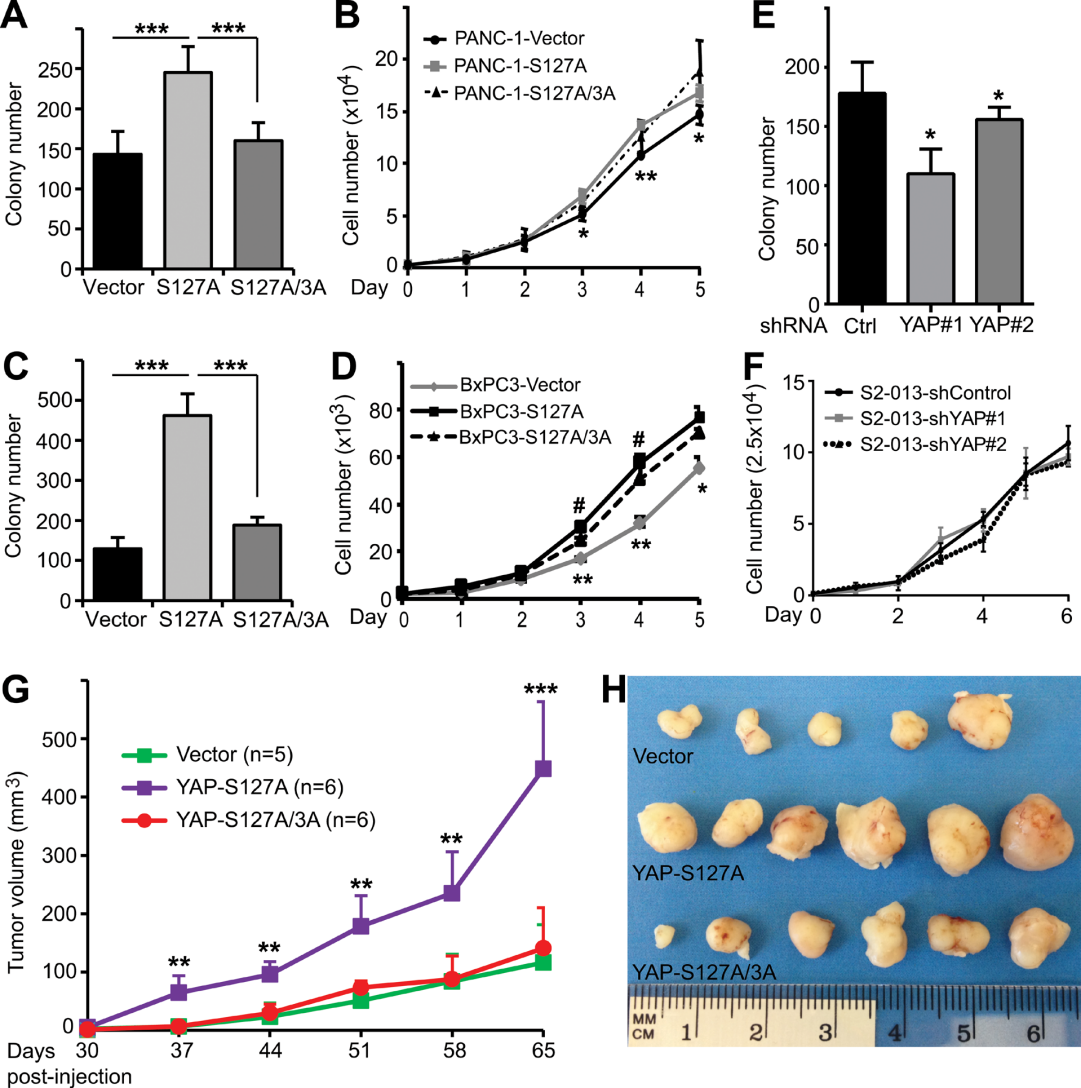
Mitotic phosphorylation promotes anchorage-independent growth and tumorigenesis of pancreatic cancer cells **A.**, Anchorage-independent growth (colony formation in soft agar) of PANC-1 cells expressing vector, YAP-S127A and YAP-S127A/3A. 3A: T119A/S289A/S367A. ***: *p* < 0.001 (*t*-test). **B.**, Cell proliferation curves for various PANC-1 cell lines with YAP overexpression. *: *p* < 0.05; **: *p* < 0.01 (YAP-S127A versus vector) (*t*-test). No significant difference between YAP-S127A and YAP-S127A/3A lines. **C.**, Anchorage-independent growth assays of BxPC3 cells expressing vector, YAP-S127A and YAP-S127A/3A. ***: *p* < 0.001 (t-test). **D.**, Cell proliferation curves for various BxPC3 cell lines with YAP overexpression. *: *p* < 0.05; **: *p* < 0.01 (YAP-S127A versus vector) (*t*-test). #: *p* < 0.05 (YAP-S127A versus YAP-S127A/3A) (*t*-test). **E.**, Anchorage-independent growth assays of S2-013 cells expressing control shRNA, or YAP shRNA. *: *p* < 0.05 (*t*-test). **F.**, Cell proliferation curves for S2-013 cell lines with control or YAP knockdown. Data are from three independent experiments and expressed as mean ± s.e.m (A-F). **G.** and **H.**, Mitotic phosphorylation is essential for YAP-S127A-promoted tumorigenesis in mice. PANC-1 cells expressing vector, YAP-S127A or YAP-S127A/3A were inoculated into athymic nude mice and tumor volume was monitored (see ‘Materials and Methods’). Tumors were also excised and photographed at the endpoint (H). **: *p* < 0.01; ***: *p* < 0.001 (YAP-S127A versus vector or YAP-S127A/3A) (*t*-test).

Recent studies showed that YAP promotes pancreatic cancer cell proliferation and anchorage-independent growth in soft agar [12, 13]. We confirmed that enhanced expression of YAP-S127A stimulated cell proliferation and anchorage-independent growth in both PANC-1 and BxPC3 cells (Figure 3A-3D). Interestingly, mutating all three mitotic phosphorylation sites to alanines greatly suppressed the activity of YAP-S127A in colony formation (Figure 3A, 3C). However, elimination of mitotic phosphorylation only moderately decreased YAP-S127A-driven cell proliferation (Figure 3B, 3D). YAP knockdown reduced colony formation in S2-013 cells (Figure 3E); however, surprisingly, S2-013 cells in which YAP was knocked down did not have an impaired proliferation rate when compared with vector-expressing S2-013 cells (Figure 3F). These data suggest that mitotic phosphorylation is also required for anchorage-independent growth, and to a lesser extent in cell proliferation, in pancreatic cancer cells.

### Mitotic phosphorylation is required for YAP-S127A-driven tumorigenesis in mice

We next evaluated the influence of YAP and its mitotic phosphorylation on tumor growth properties in animals. PANC-1-vector, -YAP-S127A and -YAP-S127A/3A-expressing cells were subcutaneously inoculated into immunodeficient mice. Every mouse in all three groups formed tumors when they reached the endpoint of the experiment. As expected, mice injected with YAP-S127A-expressing cells had significantly larger tumors at every experimental point (*p* < 0.001, YAP-S127A vs vector) (Figure 3G). Interestingly, tumors from mice harboring YAP-S127A/3A-expressing cells were much smaller when compared with those from mice inoculated with YAP-S127A-expressing cells (*p* < 0.001) (Figure 3G, 3H). These results support the hypothesis that mitotic phosphorylation is essential for active YAP-promoted pancreatic tumor growth *in vivo*.

### Upregulation and activation of YAP in metastatic tumors with PDAC

**Figure 4:**
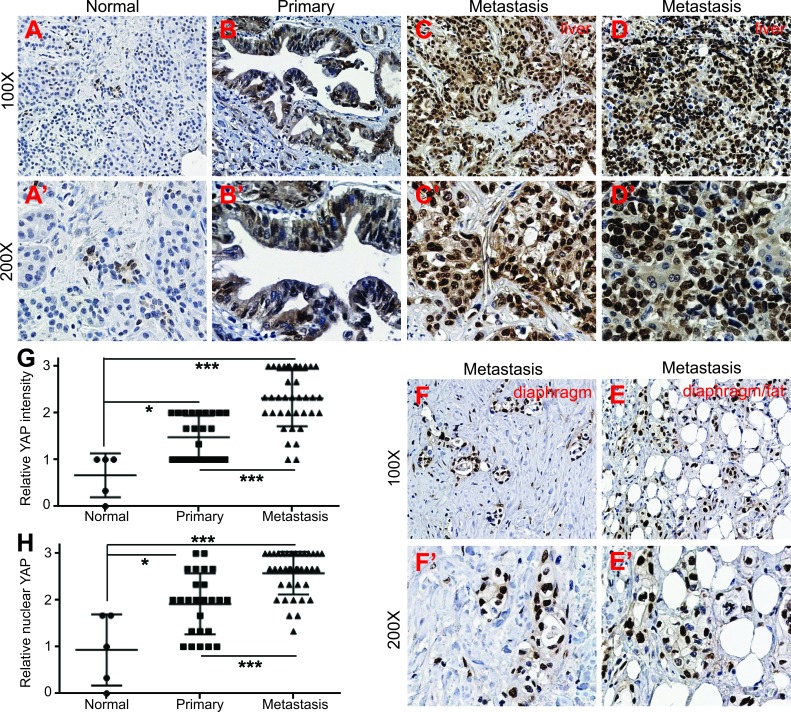
Upregulation and nuclear localization of YAP in metastatic pancreatic tumors **A.**-A', Immunostaining for YAP in normal pancreas. **B.**-B', Immunostaining for YAP in primary PDAC tumor. **C.**-**D.**', YAP staining in liver metastasis with PDAC. **E.**-**F.**, YAP staining in additional two pancreatic metastases to diaphragm/fat tissue. **G.**, Quantification of YAP IHC staining intensity in normal pancreas, pancreatic primary and metastatic tumors. **H.**, Quantification of YAP nuclear localization based on IHC staining in normal pancreas, pancreatic primary and metastatic tumors. ***: p < 0.001, **: *p* < 0.01, *: *p* < 0.05.

Previous studies, including ours, have demonstrated that YAP is overexpressed and/or hyperactivated (as shown by nuclear localization) in pancreatic primary tumor samples [11, 12]. However, it is not known to what extent YAP activity/expression correlates with the metastasis of pancreatic cancer in the clinical setting. This unknown is largely due to difficulties in obtaining high quality metastatic specimens of pancreatic cancer. The specific activity of YAP-S127A and YAP3D in cell motility and invasiveness in pancreatic cancer cells led us to investigate the correlation between YAP levels/activities and metastasis in pancreatic cancer. With access to rare matched metastatic pancreatic tumor tissue from the University of Nebraska Medical Center's Rapid Autopsy Pancreatic Program, we performed immunohistochemical staining with tissue microarrays containing normal pancreas, primary tumors and matched metastatic samples with PDAC (see ‘Materials and Methods’). Immunostaining demonstrated that overall YAP expression was very weak in all normal pancreas (Figure 4A, 4A', *n* = 5). Consistent with previous studies [11, 12], YAP levels were increased in primary tumors (Figure 4B, 4B' and 4G, *n* = 25). All of the primary tumors showed weak to moderate staining intensity and no single case had strong staining signal (Figure 4B, 4B' and 4G). Importantly, the most significant change we observed was the dramatic upregulation of YAP in metastatic samples (Figure 4C-4F', *n* = 38). Sixty percent (23/38) of metastasis showed strong staining and only 7 of the metastatic samples had weak staining (compared with more than half of the primary tumors showing weak staining) (*p* < 0.001, metastatic versus primary tumors). Furthermore, strong nucleus-localized (hyperactive) YAP staining was detected in about 70% metastatic tumors (Figure 4C-4F' and 4H, 26/38) while nuclear YAP was weak to moderate in 72% (18/25) of primary tumors (*p* < 0.001). Of note, the majority (12/14) of diaphragm and small bowel metastases showed strong nuclear-localization staining (Figure 4E-4F'). However, the lung and lymph node metastases had overall moderate YAP expression (data not shown). These data, together with our cell culture models (Figures 1 and 2), suggest that YAP functions as a novel promoter in the metastasis of pancreatic cancer in the clinical setting.

### Mitotic phosphorylation is required for YAP's transcriptional activity without affecting the binding to TEAD1

**Figure 5:**
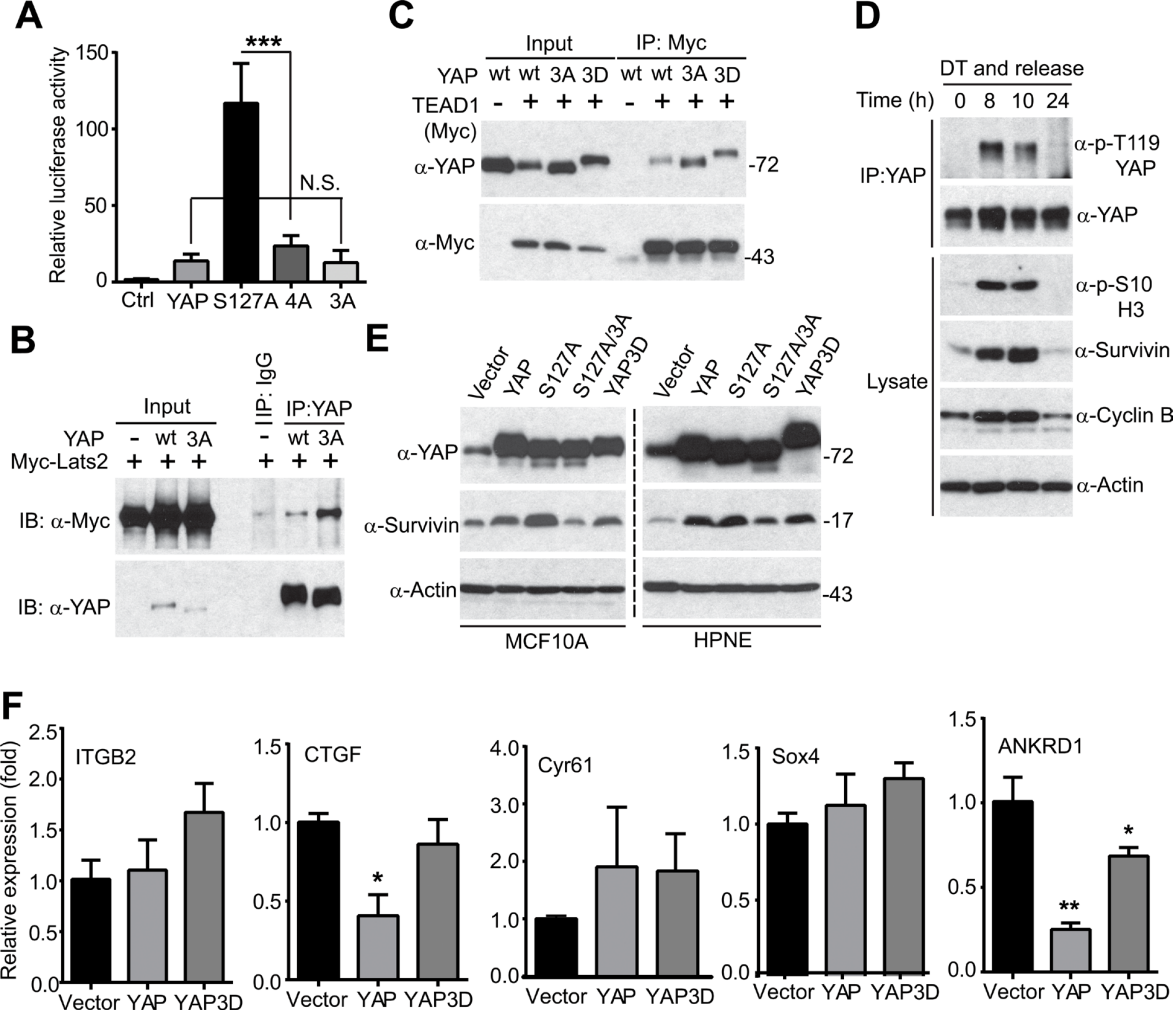
Mitotic phosphorylation is required for YAP's transcriptional activity **A.**, Luciferase reporter assays in HEK293T cells. Cells were transfected with reporters and various YAP constructs and harvested 48 hours post-transfection for measuring luciferase activity. Expression levels of YAP (and mutants) are similar (data not shown). Ctrl: control (empty vector); YAP3A: T119A/S289A/S367A; 4A: S127A/3A. N.S.: not significant. Data are expressed as the mean ± s.e.m. of three independent experiments. ***: *p* < 0.001(t-test). **B.**, Mitotic phosphorylation impacted YAP's binding to Lats2. HEK293T cell were transfected with various DNA constructs as indicated and were harvested 48 hours post-transfection for immunoprecipitation (IP). **C.**, Mitotic phosphorylation did not significantly affect YAP's binding to TEAD1. HEK293T cell were transfected with various DNA constructs as indicated and were harvested 48 hours post-transfection for IP. **D.**, HeLa cells were synchronized by a double thymidine (DT) block and release method. Endogenous YAP was immunoprecipitated at the indicated time points and probed with the indicated antibodies. Total cell lysates before IP were also analyzed to confirm the cell phase status. **E.**, HPNE cells expressing vector, YAP, YAP-S127A, YAP-S127A/3A (YAP4A) or YAP3D were probed with the indicated antibodies. **F.**, Quantitative RT-PCR of known YAP targets in HPNE cells. Data are expressed as the mean ± s.e.m. of three independent experiments. **: *p* < 0.01; *: *p* < 0.05 when compared to vector control (*t*-test).

YAP is a transcriptional co-activator, and the TEAD1-4 transcription factors are the primary mediators of YAP in the Hippo pathway [15]. We determined whether mitotic phosphorylation affects YAP's transcriptional activity using luciferase reporter assays. As shown in Figure 5A, YAP3A (non-phosphorylatable mutant) has similar activity as wild type YAP (Figure 5A), suggesting that CDK1-mediated phosphorylation did not alter the basal transcriptional activity of YAP/TEAD under this condition. Interestingly, mutating phosphorylation sites to alanines greatly suppressed YAP-S127A's activity, suggesting that mitotic phosphorylation became essential only when YAP was hyperactivated (e.g. when upstream tumor suppressors are deregulated) (Figure 5A, compare 4A to S127A). YAP is phosphorylated and inactivated by Lats1/2 kinases [11, 16] and binds to TEAD transcriptional factors [15] to control target gene expression. We further determined whether mitotic phosphorylation affects YAP's association with these factors. As shown in Figure 5B, the non-phosphorylatable YAP3A mutant possessed stronger binding affinity with Lats2 than wild type YAP. However, mitotic phosphorylation did not alter the association between YAP and TEAD1 (Figure 5C).

YAP induces many targets including CTGF (connective tissue growth factor), survivin, Cyr61 (cysteine-rich angiogenic inducer 61), ITGB2 (integrin beta 2), ANKRD1 (ankyrin repeat domain-containing protein 1), and Sox4 (SRY-related HMG-box 4). Interestingly, mitotic phosphorylation of YAP was coincident with the elevated levels of survivin (Figure 5D). We previously showed that YAP/YAP-S127A induced survivin expression in HPNE cells [11]. We confirmed this observation in both MCF10A and HPNE cells (Figure 5E). Importantly, mutating mitotic phosphorylation sites to alanines greatly suppressed YAP-S127A-induced survivin expression (Figure 5E, compare S127A/3A to S127A), suggesting that mitotic phosphorylation was essential for YAP-S127A to induce survivin expression in immortalized epithelial cells (Figure 5E). However, surprisingly, CTGF, Sox4, ANKRD1 and ITGB2 were not induced by YAP or YAP3D in HPNE cells (Figure 5F), while their expression was greatly upregulated by YAP in breast epithelial cells and in the mouse liver [11, 15]. Of note, though survivin was induced in HPNE cells, wild type YAP induced survivin expression as well as YAP3D did (Figure 5E, compare YAP3D to YAP). Considering that phospho-mimetic YAP mutant (YAP3D) possesses much higher activity in migration and invasion compared to wild type YAP (Figure 1), we excluded these known targets as downstream effectors in mediating YAP phosphorylation-promoted motility and invasion. Together, these observations suggest that YAP3D controls specific targets involved in motility and invasion in pancreatic cells.

### LPAR3 mediates YAP3D-driven migration and invasion

**Figure 6:**
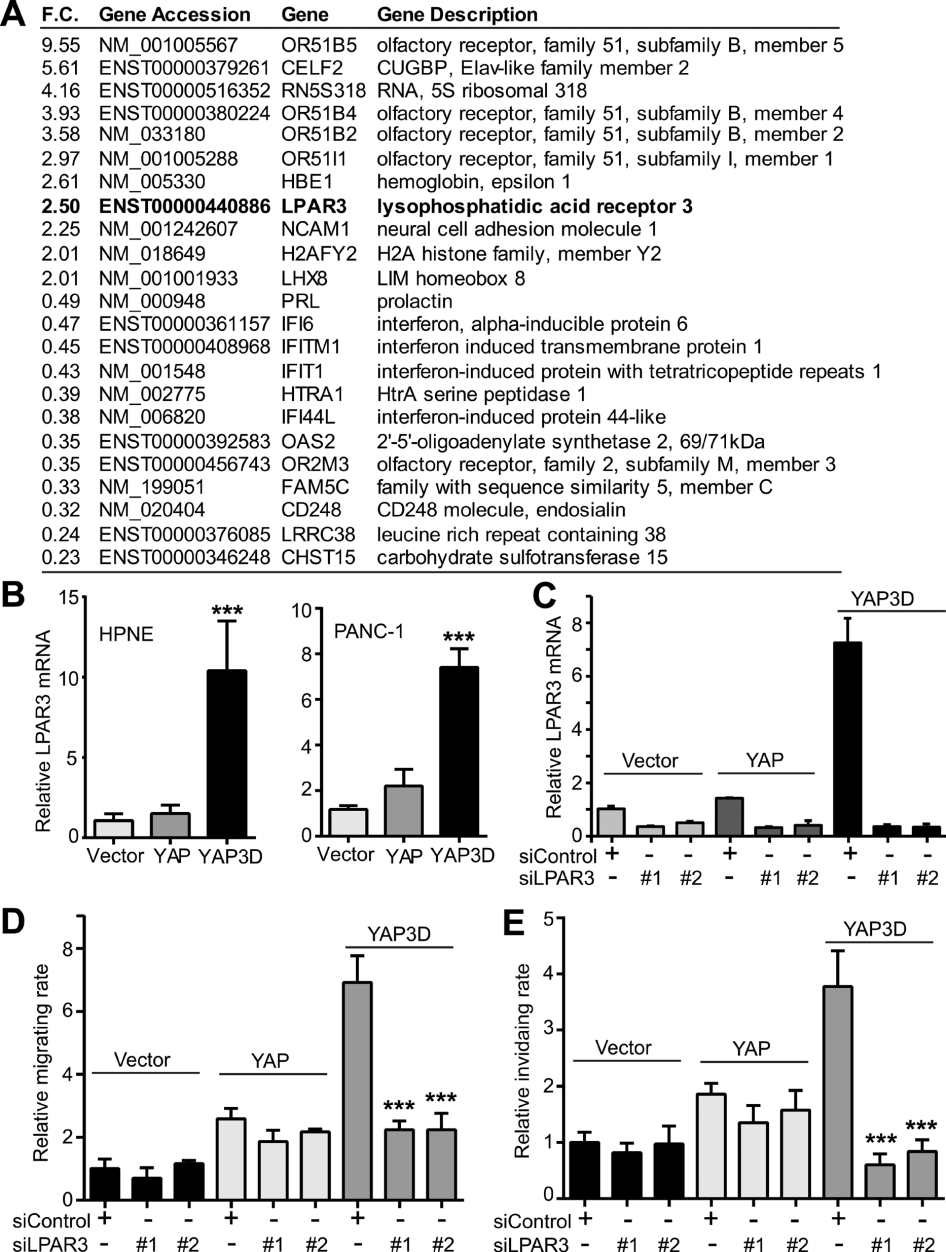
Identification of LPAR3 as a potential target of hyperactive YAP **A.**, A partial list of up- or down-regulated genes from genome-wide expression analysis (HPNE-vector versus HPNE-YAP3D). F.C.: fold change. **B.**, Quantitative RT-PCR confirmation of LPAR3 mRNA in HPNE and PANC-1 cells. Data are expressed as the mean ± s.e.m. of three independent experiments. ***: *p* < 0.001 when compared to vector control and YAP (t-test). **C.**, Quantitative RT-PCR confirms the siRNA-mediated downregulation of LPAR3 mRNA in HPNE cells expressing vector, YAP or YAP3D. **D.**-**E.**, LPAR3 mediates YAP3D-driven migration (D) and invasion (E). Migration and invasion assays in HPNE-vector, -YAP, and -YAP3D cells with control siRNA or LPAR3 knockdown were done as in Figures 1 and 2. Data are expressed as the mean ± s.e.m. of three independent experiments. ***: *p* < 0.001 when compared to control siRNA transfection (*t*-test).

The specific activity of YAP3D (and YAP-S127A) in the motility of pancreatic cancer cells prompted us to identify the target(s) that mediates cell migration and invasion. We have performed a genome-wide gene expression analysis in HPNE-vector and -YAP3D cells (Figure 6A). The gene LPAR3 (lysophosphatidic acid receptor 3) attracted us since LPAR3 was recently identified as one of the potential Hippo pathway receptors [17] and found to promote cell migration and invasion [18, 19]. Quantitative RT-PCR confirmed that LPAR3 mRNA was greatly induced by YAP3D, but not by wild type YAP or vector in both HPNE and PANC-1 cells (Figure 6B). To assess the functional relevance of LPAR3 in motility, control siRNA or siRNA targeting LPAR3, was transfected into HPNE-control, -YAP, and -YAP3D cells, and cell migration and invasion were analyzed. Introduction of two independent siRNAs efficiently knocked down LPAR3 in HPNE-vector, -YAP and -YAP3D cells (Figure 6C). Importantly, LPAR3 knockdown greatly impaired YAP3D-driven migration and invasion (Figure 6D, 6E). The migratory and invasive abilities were not significantly altered by LPAR3 knockdown in HPNE-vector and -YAP cells (Figure 6D, 6E). These data suggest that mitotic phosphorylation (YAP3D) promotes migration and invasion by specifically upregulating LPAR3 expression in pancreatic cells.

## DISCUSSION

The role of YAP in cell proliferation and apoptosis has been extensively studied in various types of cancer cells; however, it is less clear regarding the function of YAP in cell motility. One of the significant findings in this study is the hyper-activity of YAP (YAP-S127A and YAP3D) in cell motility and invasiveness in pancreatic cancer cells (Figures 1-3). Cell migration and invasion are important processes for metastatic tumor formation [20-22]. Consistent with these observations, we demonstrate that YAP expression is significantly upregulated and activated (revealed by nuclear localization) in metastatic samples with PDAC compared with primary tumors (Figure 4). These data suggest that YAP functions as a positive regulator in cancer progression and metastasis in addition to its ability to cause oncogenic transformation in pancreatic cancer. Hippo-YAP signaling is deregulated in many human malignancies and is a potential target for cancer therapy [5-7]. Indeed, several recent studies from animal models strongly support the feasibility of targeting YAP (using Verteporfin as an inhibitor of YAP/TEAD complex) in cancer [23, 24]. Interestingly, Verteportfin-based photodynamic therapy overcomes Gemcitabine resistance in pancreatic cancer cells [25]. Thus, these studies support the use of Verteporfin as a potential pharmacologic approach to inhibit YAP signaling in the context of pancreatic cancer.

In this study we provided evidence that active YAP and its mitotic phosphorylation is essential in cell motility; however, the underlying mechanisms need to be further explored. Previous studies have linked the mitotic machinery and microtubule cytoskeleton to cancer cell migration and invasion [26]. It will be interesting to investigate whether active YAP (YAP-S127A and YAP3D) has a direct connection with the mitotic apparatus and/or microtubules, and how YAP modulates these dynamics to facilitate in cell motility. Furthermore, it is also important to investigate the CDK1 phosphorylation status of YAP and to determine the correlation between CDK1/cyclin B activity and YAP phosphorylation in pancreatic cancer patients. Interestingly, CDK1 overexpression or elevated cyclin B expression is often observed in human cancers especially in melanoma [27-29]. Whether CDK1/cyclin B expression/activity is increased in pancreatic cancer remains to be determined.

YAP targets are largely cellular context-dependent. Several known targets including CTGF, Sox4, Cyr61 and ANKRD1 were not induced by YAP in pancreatic epithelial cells (Figure 5). Another interesting finding in the current study is the unique transcriptional program of YAP3D in immortalized pancreatic cells (Figure 6). While YAP3D robustly induced LPAR3 expression, wild type YAP was not sufficient to do so. Interestingly, YAP-S127A was also able to induce LPAR3 expression in immortalized and cancer pancreatic cell lines (S. Y. and J. D., unpublished observations). LPAR3 was not induced by YAP/YAP-S127A in the mouse liver and mammary epithelial cells [11, 16]. It is currently unknown how this specificity is achieved. Further experiments are also needed to determine whether LPAR3 is a direct target of YAP.

We believe the identification of LPAR3 as a downstream mediator for active YAP-driven migration and invasion is of importance since LPAR3 is a G-protein coupled receptor, and G-protein coupled receptors are demonstrated targets for almost half of all drugs [30]. Importantly, LPA and LPARs have been implicated in multiple functions in human malignancies including ovarian and breast cancers, especially in cell motility and invasion [18, 31]. For example, overexpression of LPAR3 and ATX (autotaxin, the major enzyme that generates LPA) promoted tumorigenesis, invasion and metastasis in a mammary transgenic mouse model [19]. LPA levels were increased in ascites of patients with ovarian cancer [32]. We are currently investigating the functional significance of the ATX-LPA-LPAR3 axis and other LPARs in pancreatic cancer. It is also worth determining the correlation between the levels of YAP (and/or the localization of YAP) and LPAR/ATX in clinical samples. These future studies may not only provide novel insights into the pathogenesis of pancreatic cancer, but also identify ATX-LPAR3 axis as a target for the treatment of invasive pancreatic cancer.

## MATERIALS AND METHODS

### Expression constructs

The pcDNA-YAP expression construct has been described [11]. Retroviral wild type YAP and YAP mutant constructs have been described [14]. Myc-Lats2 was described in [33]. Myc-TEAD1 DNA and lentiviral shRNAs against human YAP were purchased from Addgene [15]. Lentivirus packaging constructs (psPAX2 and pMD2.G) were also from Addgene. Point mutations were generated by the QuikChange Site-Directed PCR Mutagenesis Kit (Stratagene) and verified by sequencing.

### Cell culture and transfection

HEK293T, HeLa, PANC-1, and BxPC3 cell lines were purchased from American Type Culture Collection (ATCC). The cell lines were authenticated at ATCC and were used at low (< 25) passages. The immortalized pancreatic epithelial cells (HPNE) were provided by Dr. Michel Ouellette (University of Nebraska Medical Center), who originally established the cell line [34] and the cells were cultured as described [11]. HEK293T and HeLa cell lines were maintained in DMEM media (high glucose, Hyclone) supplemented with 10% FBS and L-glutamine plus 100 units/ml penicillin and 100 μg/ml streptomycin (Invitrogen). PANC-1, BxPC3, Colo357 and S2-013 cell lines were maintained in RPMI-1640 media (ATCC) supplemented with 10% FBS and 100 units/ml penicillin and 100 μg/ml streptomycin. Attractene and HiPerFect (Qiagen) were used for transient overexpression and siRNA transfections, respectively, following the manufacturer's instructions. YAP and LPAR3 siRNA oligonucleotides were synthesized by GenePharma based on the following target sequences YAP#1: 5′-caggtgatactatcaaccaaa-3′; YAP#2: 5′-gaccaatagctcagatccttt; LPAR3#1: 5′-gcctatgtattcctgatgttt-3′; LPAR3#2: 5′-ggagaggcacatgtcaatcat-3′. All other chemicals were either from Sigma or Thermo Fisher.

### Retrovirus and lentivirus packaging and infection

Ectopic expression of YAP or mutants in HPNE, BxPC3 and PANC-1 cell lines was achieved by a retrovirus-mediated approach. Retrovirus packaging and infection were done as we have described previously [33]. The transduced cells were then selected with 600 μg/ml of neomycin (at 48 hours post-infection) to establish stably expressing YAP or YAP mutant cell lines. YAP downregulation in S2-013 and Colo357 cells was obtained by lentivirus-mediated YAP shRNA expression. Lentivirus generation and infection were performed as described with slight modifications [15]. The transduced cells were then selected with puromycin (5 μg/ml for S2-013 cells and 1 μg/ml puromycin for Colo357) to establish cell lines in which YAP was stably knocked down.

### Quantitative real time PCR

Total RNA isolation, RNA reverse transcription and quantitative real time-PCR were done as we have described previously [33]. Primer sequences for LPAR3 are as follows: LPAR1: ccaggagtccagcagatgat (forward); gtctcggcatagttctgga (reverse); LPAR2: cagcctaaaccatccaggag (forward); cagcctggtcaagactgttgt (reverse); LPAR3: gggtccagcataccacaaac (forward); LPAR3: caacgtcttgtctccgcata (reverse).

### Luciferase reporter assay

Luciferase reporter assays were performed in 24-well plates in HEK293T cells. 8XGTIIC-Luciferase (Addgene 34615, [35]), SV40-Renilla (Addgene 27163, [36]) and indicated plasmid (empty vector, wild type YAP or YAP mutant construct) were co-transfected in triplicate as we have described previously [11]. Luciferase activity was assayed at 48 hours post-transfection by the Dual-luciferase reporter assay system (Promega) following the manufacturer's instructions.

### Antibodies, immunoprecipitation and western blot analysis

The YAP antibodies from Abnova (H00010413-M01) and Abcam (52771) were used for immunoprecipitation of endogenous YAP and for Western blotting, respectively, throughout the study. Rabbit polyclonal phospho-specific antibody against YAP T119 has been previously described [14]. Anti-β-actin, anti-Myc, and anti-cyclin B antibodies were from Santa Cruz Biotechnology. Anti-phospho-S10 H3 and anti-survivin antibodies were from Cell Signaling Technology. Immunoprecipitation and Western blotting assays were done as previously described [37].

### Cell proliferation and anchorage-independent assays

For cell proliferation assays, 3000 cells were seeded in 24-well plates in triplicate. Cells were counted by a hemocytometer from days 1-6 and proliferation curves were made based on the cell number of each well from three independent experiments. Soft agar colony formation assays were conducted in 6-well plates as we have described [11].

### Cell migration and invasion assays

*In vitro* analysis of invasion and migration was assessed using the BioCoat invasion system (BD Biosciences) and Transwell system (Corning), respectively, according to the manufacturer's instructions. The cells were trypsinized and resuspended in the medium without serum and/or growth factor. Cell suspension (containing 5000 cells) was added to the insert and incubated for 18 hours at 37°C. The invasive and migratory cells were fixed with 3.7% PFA and stained with ProLong^®^ Gold Antifade Reagent with DAPI. The relative invading and migrating rate were calculated as we have previously described [14, 38].

### Microarray analysis

Total cellular RNA from the HPNE-vector and HPNE-YAP3D (mitotic phosphorylation-mimetic mutant) stable cell lines was extracted using TRIzol reagent and further purified by the RNeasy kit (QIAGEN) following the manufacturer's instructions (Invitrogen). RNA was processed using the RNA amplification protocol described by Affymetrix and 10 μg of total fragmented cRNA were hybridized to the GeneChip arrays (Affymetrix human 2.0). Affymetrix slide hybridization, image analysis, and data analysis were done as we have previously described [11]. Hybridization and raw data analysis were done by the Microarray Core Facility at the University of Nebraska Medical Center. All Affymetrix data from different samples were normalized and summarized with the robust multi-average (RMA) method [39] implemented in the Affymetrix Expression Console. The LIMMA [40] method was used to compare the gene expression between the experimental and control samples. The Benjamini Hochberg method [41] was used to control the false discovery rate to be no more than 0.05. The genes with Benjamini Hochberg adjusted p value less than 0.05 and at least 1.5 (or 1/1.5) fold changes between groups were declared to be differentially expressed.

### Immunohistochemistry (IHC) staining

Human pancreatic, metastatic, and unaffected specimens from decedents who have previously been diagnosed with pancreatic ductal adenocarcinoma were obtained from the University of Nebraska Medical Center's Tissue Bank through the Rapid Autopsy Pancreatic (RAP) program in compliance with IRB 091-01. To ensure specimen quality, organs were harvested within three hours post mortem and the specimens flash frozen in liquid nitrogen or placed in formalin for immediate fixation. Sections were cut from paraffin blocks of formalin fixed tissue into 4 micron thick sections and mounted on charged slides [42]. Two tissue microarray (TMA) slides consisting of 68 cases with normal (*n* = 5), primary (*n* = 25) and matching metastatic samples (*n* = 38 including 22 liver, 10 diaphragm, 4 small bowel, 1 lung and 1 lymph node metastases) were used. Slide deparaffinization, antigen retrieval, blocking, and anti-YAP antibody (Cell Signaling 4912, at 1:100 dilutions) staining were performed as we have described [11]. Cell nuclei were stained with Hematoxylin. Ventana iScan HT (Roche) was used for slide scanning with a 20X objective lens at the Department of Pathology, University of Nebraska Medical Center. The staining results were independently evaluated by three researchers including one pathologist (K. F.). Both the YAP staining intensity (a scale of 0 to 3 was used: 0-negative, 1-weak, 2-moderate, and 3-strong) and nuclear localization (the percentage of tumor cell nuclei stained, 0-no staining, 1-≤10%, 2-10-50%, and 3->50%) were scored [43].

### Animal studies

For *in vivo* xenograft studies, PANC-1 cells expressing vector, YAP-S127A (hyperactive mutant) and YAP-S127A/3A (YAP4A, a mitotic phosphorylation-deficient mutant) (1.5×10^6^ cells each line) were subcutaneously injected into the left flank of 6-week-old female athymic nude mice (Ncr-nu/nu, NCI). Five (vector) or six (YAP-S127A and YAP4A) animals were used per group. Tumor sizes were measured once a week using an electronic caliper when tumors in the vector/control group are visible. Tumor volume (V) was calculated by the formula: V= 0.5 x length x width^2^ [11]. Mice were euthanized at 9 weeks post-injection and the tumors were excised for subsequent analysis. The animals were housed in pathogen-free facilities. All animal experiments were approved by the University of Nebraska Medical Center Institutional Animal Care and Use Committee.

### Statistical analysis

Data were analyzed using a two-tailed, unpaired Student's *t*-test. The IHC intensity and localization scores were summarized using median and inter-quartile-range (IQR) for normal, primary and metastasis samples separately, and compared among groups using the nonparametric Kruskal Wallis test. When the comparison yielded a significant p value, the pair-wise comparisons with Bonferroni method for multiples comparisons were conducted. A *P* value of < 0.05 was considered as indicating statistical significance.
